# Suggestions on Relieving Physical Anxiety of Medical Workers and Improving Physical and Mental Health Under the COVID-19 Epidemic—A Case Study of Meizhou City

**DOI:** 10.3389/fpubh.2022.919049

**Published:** 2022-06-17

**Authors:** Zhi-Cheng Lu, Chien-Hsing Tseng, Hsiao-Hsien Lin, Yuan-Shing Perng, Yi-Han Tseng

**Affiliations:** ^1^School of Physical Education, Jiaying University, Meizhou, China; ^2^Department of Recreation and Sport Management, Shu-Te University, Kaohsiung, Taiwan; ^3^Department of Leisure Industry Management, National Chin-Yi University of Technology, Taichung, Taiwan; ^4^Department of Forestry, National Chung Hsing University, Taichung, Taiwan; ^5^Department of Tourism Leisure and Health Management, Chung Chou University of Science and Technology, Yuanlin City, Taiwan

**Keywords:** SARS-CoV-2, healthcare professionals, environment risk, body anxiety, leisure satisfaction, physical and mental health

## Abstract

The study examined the effects of swimming pools on healthcare professionals' willingness to engage in recreational activities, physical anxiety, and physical and mental well-being in the context of COVID-19. The research adopted the mixed research method, used SPSS 26.0 statistical software to test the reliability of the questionnaire, and then collected 840 valid questionnaires; first analyzed the data with basic statistics, *t*-test, ANOVA, and PPMCC test methods, and then used the interview method to collect expert opinions. A multi-check approach assembled all data and discussions. The study found that the use of personnel dynamic tracking systems or measures, combined with sodium hypochlorite and repeated filtration to stabilize water quality, could maintain the confidence of most medical workers in the swimming pool sports environment for epidemic prevention and avoid violations. The government could formulate safety prevention and control mechanisms in traffic and establish appropriate traffic routes. Next, formulated a prescription for swimming or other physical activity mechanisms for men aged 31–50 and redesigned measures for medical staff over 51 years old to have tense head issues and physical fatigue, promote blood circulation and improve sleep quality. This will promote the purpose of relieving stress and regulating the physical and mental health of medical staff after engaging in swimming.

## Introduction

Since the outbreak of COVID-19 in December 2019, humanity has been facing one of the most difficult times in recent global epidemic cases and is still not free from the threat (1). In recent years, governments around the world have vigorously promoted vaccine management policies, invested a lot of medical resources, and hoped that the popularization of vaccines would increase people's resistance (2). Governments actively call on the people to wear masks, wash their hands frequently, avoid gatherings and talk closely to avoid being infected by the virus and even spreading the epidemic (3). In recent years, a number of studies have also proposed ways to teach people how to maintain regular exercise to maintain health in the context of COVID-19 (4–9). It is expected that exercise can improve physical fitness, enhance human immunity, relieve the pressure of medical staff, achieve the goal of resisting viruses, and enhance individual physical and mental health (10).

Because SARS-CoV-2 is an enveloped virus, it is highly infectious, hydrophilic, and difficult to eradicate (11). And the virus gene gradually adapts to the human body, and the virus gene is constantly mutated (12), which increases virus transmission efficiency (13). However, the epidemic threat still exists today, and the environment is full of infection risks, which hinders the willingness to go out (14). As a result, people are full of insecurity in the social environment (10, 14), causing people to panic, anxiety, loss and other psychological pressures, affecting their physical health (3, 15). So far, there have been 513 million cases of infection in 199 countries around the world, with 6.23 million deaths, and infected people continue to be found (16). This phenomenon has impacted the medical systems of various countries, seriously the workload of medical staff and exhausting their physical and mental health (17). Therefore, some scholars believe that finding ways to improve the physical and mental stress of medical staff, provide a safe leisure environment, and promote physical and mental health will be a good way to maintain the service quality of medical staff and stabilize national public health management (10, 18). So we think this is an important research topic at present.

Swimming has the functions and characteristics of enhancing cardiopulmonary function, improving physical fitness, and improving physical and mental health (19, 20). Many countries actively build swimming pools to provide a safe and hygienic recreational sports environment, allowing people to continue swimming to maintain health (21, 22). In order to maintain the sanitation and cleanliness of swimming pool water, sodium hypochlorite has been widely used for water disinfection and environmental cleaning for a long time (21). This method has been recognized by the World Health Organization and the National Centers for Disease Control and Prevention and stated that if the effective residual chlorine in the swimming pool can be maintained above 1 ppm, it can effectively inhibit Escherichia coli and Legionella (22, 23), and decompose bacterial nuclei (24). This measure is applied to enveloped virus-type pathogens such as SARS-CoV-2. As long as the concentration of sodium hypochlorite is increased to 0.5% (5,000 ppm), highly resistant pathogens can be effectively inhibited (25–28). In addition, through effective filtration facilities, impurities in swimming pool water are repeatedly filtered to maintain the hygiene of water quality (29, 30), and sodium hypochlorite is used for environmental disinfection (27, 28). Some scholars believe these measures will provide swimming pools with a safe and hygienic exercise environment during the epidemic (25–30). Therefore, we believe that when the surrounding environment of the swimming pool and the quality of the water are guaranteed, it should be possible to create a safe leisure sports environment (31, 32). Therefore, if the medical staff is advised to go swimming in swimming pools, they should be able to obtain regular exercise, enhance cardiopulmonary function, improve physical fitness, reduce anxiety, and stabilize physical and mental health under a low-risk awareness of the exercise environment (20, 21).

However, since the current problem is not under control, there has always been an infection risk in the surrounding environment (15), which seriously affects people's willingness to go out. Therefore, there is a gap between plan and reality (8, 10). Therefore, some scholars suggest that it is necessary to understand the risk perception of the current swimming pool by medical staff engaged in swimming, as well as the changes in personal anxiety, exercise benefits, and physical and mental health feelings after going to exercise (32). However, these changes and experiences take time (33, 34). Through the awareness of environmental risk assessment, people can assess the potential crisis and risk degree of the surrounding environment (35). Through the perception of physical anxiety, medical staff's personal anxiety changes after engaging in swimming can be found (32). Through the perception of leisure satisfaction, we can find the changes in personal leisure satisfaction after medical staff engage in swimming (36). Finally, the physical and mental health perception can be used to understand the improvement of personal physical and mental health after medical staff engages in swimming (37–39). These answers can be corroborated by participants' feelings (34, 40, 41). Therefore, we believe that by discussing environmental risks, physical anxiety, leisure satisfaction will effectively improve the environmental risk perception of medical personnel, reduce anxiety, improve leisure benefits, and achieve the goal of promoting physical and mental health, and physical and mental health perception, we can know whether the physical and mental health of medical staff can be improved after swimming.

Furthermore, after analyzing the literature, we know that although the current research directions on COVID-19 and human health problems are quite extensive (1, 2, 4, 5, 17). However, most of them discussed the physical and mental health pressure and epidemic prevention measures during the epidemic (5, 25–30, 42). Although the impact of participating in tourism activities during the epidemic has also been discussed (33, 36, 43, 44), methods to improve people's health benefits have also been proposed, such as Qigong (6), intermittent fitness exercises (7), mountain climbing (4), and jogging (8). More recently, related impacts of leisure, travel, living and working environment, physical and mental health have also been discussed (36–38), as well as environmental risks, satisfaction, and physical and mental health issues (40, 41, 45). However, the researchers found that swimming was not discussed as a topic, and the environmental risks, physical anxiety, leisure satisfaction, and physical and mental health perceptions of healthcare workers engaged in swimming in SARS-CoV-2 settings.

Based on the above descriptions, we believe that taking the epidemic period as the timeline, and collecting the opinions of medical staff who continue to swim during the epidemic from the aspects of environmental risk, physical anxiety, leisure satisfaction, and physical and mental health cognition, the truth will be obtained. These answers will help the government, medical institutions, or medical staff finds a safe exercise environment and regular exercise methods, provide effective exercise, improve resistance, maintain medical quality, and stabilize public health orders during the COVID-19 epidemic.

## Literature Review

### Leisure Environment Risk

Risk refers to the potential factors that people may develop adversely, personally, or financially (35). Sports environmental risk is mainly to understand the unpredictable but potentially far-reaching potential problems that arise when people or society face the environmental hazards of the epidemic (46). Risk causes are often at odds with everyday knowledge, even conflicting or unpredictable (47). The individual's reaction to risk is the environmental risk perception (48).

Establishing a risk requires three elements: uncertainty, the possibility of loss, and future attributes (49). Individuals feel uncertain about future outcomes, and the outcome may have a negative impact, and the higher the uncertainty, the greater the risk (50). Some scholars believe that predicting risks can usually be discussed in terms of behavior, facility and environmental safety, potential risks, economic losses, and increased social costs (48–51).

Therefore, the researchers believe that the current situation of swimming pools should be referred to as environmental risk prediction indicators. From the aspects of exercise mode, equipment safety, movement fluency, potential risks, happiness, and social costs, it is possible to understand the environmental risk perception of the public when exercising in the swimming pool.

### Physical Anxiety

Anxiety refers to an individual's state of excessive, widespread, persistent, and perceived uncontrollable worry about many events or activities, almost most of the time, for at least the past 6 months (52). Anxiety assessment mainly aims to understand people's current psychological mood and distress from different aspects and provide suggestions to improve the quality of life (53). Body anxiety refers to an individual's negative subjective perceptions and feelings that stimulate the body with the environment or time (54).

The establishment of anxiety requires a pathogenic context. The operation process of a specific system, such as cognition related to information processing, emotional stimulus processing, coping, and psychophysiological aspects, are key factors (55). If the individual is worried about the phenomenon or event that continues to occur, or even a catastrophic phenomenon occurs, it may cause psychological trauma (56). Some scholars believe that physical anxiety can be assessed from the aspects of physiological behavior, psychological status, behavioral performance, and effectiveness judgment (57).

Therefore, researchers believe that the answer should be obtained based on the physical anxiety state that the research subjects may face and then referring to relevant literature on physical anxiety, psychological behavior settings (from motor behavior anxiety, tension), physiological behavior (from limb stiffness, muscle tension, stomach tension, rapid heartbeat, and other behavior), and effectiveness judgments (from the affirmation of athletic performance, achievement of expected goals, exceeding levels, and zero mistakes) to predict the state of physical anxiety.

### Leisure Satisfaction

Leisure satisfaction refers to the positive perceptions of individuals engaged in leisure activities after comparing their perceived leisure experience with the actual environment (58). To put it simply, people judge according to the current leisure experience whether the acquired individual needs and satisfaction meet the expected goals, which is leisure satisfaction (43).

Leisure satisfaction uses personal activity experience to compare prior experience, personal expectations, or expectations (59). When the actual leisure environment or activity content conforms to the individual's expectations, a satisfied attitude is generated in the heart (58, 59). The higher the satisfaction, the higher the positive energy cognition obtained (60). The more satisfaction one can obtain from personal psychological needs, the more satisfied with the current life and feelings, and the more willing to revisit (58). Some scholars believe that although the influencing factors of leisure satisfaction can be divided into internal and external interference (61), it can be discussed in terms of decision-making measures, environment, facilities, and personal leisure benefits (59–61).

Therefore, researchers believe that the answer should be obtained with reference to the literature on leisure satisfaction and according to the problems that the research topic may encounter, such as decision-making measures that maintain a safe distance, people wearing masks, keeping track of personnel movements, and the environment and facilities being discussed in terms of personal hygiene of swimmers, clean water, and clean facility environment.

### Cognition of Physical and Mental Health

Physical and mental health refers to people's physical, psychological, and social aspects to achieve a state of well-being (59). It can present the actual situation through scientific testing evidence such as self-assessment (58), which belongs to the analysis method of self-perception assessment (60). When the public perceives a higher health risk, the power to influence an individual's final behavioral decision will be greater (60).

Physical and mental health cognition mainly investigates the current state of individual physical and mental health and presents the impact of the current environment on people (62). Some scholars believe that physical and mental health can be divided into four levels: mental, spiritual, physical, and negative attitudes (60, 61). And people can get answers from 16 questions such as learning, fear, performance, lack of interest in things, lack of time, headache, fatigue, back pain, insomnia, stomach pain, overeating, muscle stiffness, tantrums, loss, burnout, depression, and suicide (60–63).

Therefore, the researchers believe that the answers should be obtained by referring to those mentioned above in physical and mental health-related literature, according to the psychological, spiritual, physical, and negative attitudes and other aspects, and with the evaluation based on 16 indicators, such as learning, fear, performance, lack of interest in things, and lack of time.

## Methods

### Framework

**Figure 1:**
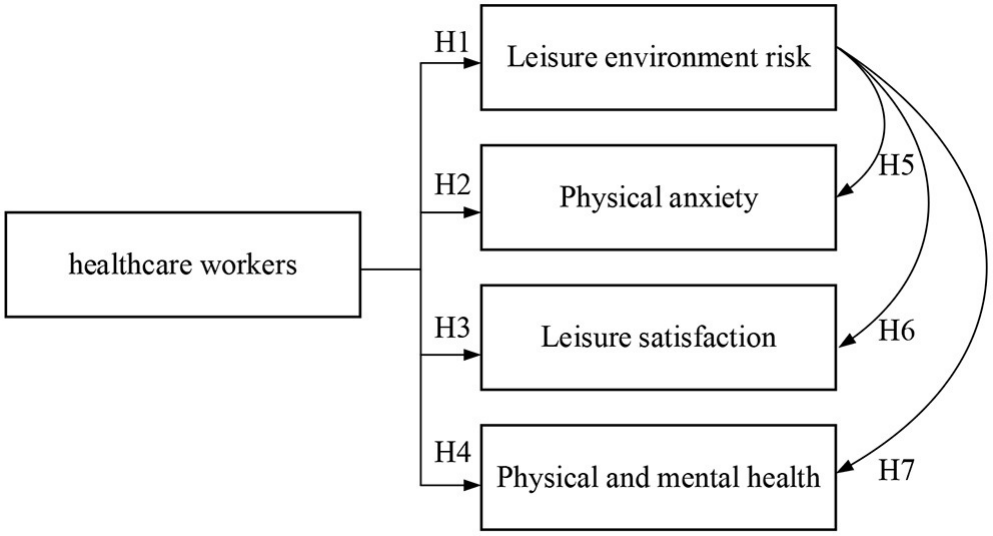
Research framework.

The study first collected relevant literature, analyzed the influence of swimming pool disinfection and filtration management measures on epidemic prevention and control, and referred to literature on environmental risks, physical anxiety, leisure satisfaction, and physical and mental health cognition. Took the epidemic period as the timeline, targeted those who continue to engage in swimming, and based on the literature on environmental exercise risk (46–51), physical anxiety (52–57), leisure satisfaction (58–61), and physical and mental health cognition (58–63), the study was summarized and analyzed. Looking forward to knowing whether swimming sports and venues would have the effect of providing people with a healthy and safe leisure environment, reducing personal anxiety, and promoting physical and mental health. The research structure is shown in Figure 1.

### Process and Analysis

At present, the research direction on COVID-19 and human health issues has been quite broad (1–30). However, there has been no discussion about the environmental risks, physical anxiety, leisure satisfaction, and physical and mental health cognition of people engaging in swimming in the context of SARS-CoV-2 with swimming as the theme. Therefore, mixed research methods were used through the feelings of actual participants (33, 34, 40, 44), quantitative research methods were used to supplement the research breadth (64, 65), and qualitative research methods were used to increase research depth (66). Then the research would be discussed in a multi-check method (67–69), which could make up for the shortcomings of research methods or insufficient theory (70).

Therefore, the researchers first referenced literature on environmental risk (46–51), physical anxiety (52–57), leisure satisfaction (58–60), and physical and mental health perceptions (60–63), and then developed a questionnaire tool. First, SPSS 26.0 statistical software was used to confirm the framework of the formal questionnaire tool with basic statistical verification and EFA verification method, and then three experts with expertise in public health, leisure sports, and decision analysis were invited to check the content validity to confirm the formal questionnaire. In November 2021, samples were collected by interest sampling method and snowball sampling method, and 840 questionnaires were finally obtained.

The data were analyzed by basic statistics, *t*-test, ANOVA, and PPMCC test. Then, the semi-structured interview method was used to collect the opinions of officials, experts, and the public according to the results of the questionnaire analysis. Next, all the data in a rigorous was collected in an orderly and logical manner and then valuable information through summarizing, organizing, and organizing methods were summarized (69). Finally, the research was carried out through the multi-check method in a multi-data and multi-perspective way (67, 68, 71).

### Hypothesis

Due to the impact of the potential risk of epidemic infection around the world, people's willingness to go out for leisure and activities has been declined (13). As many studies had confirmed that swimming pool disinfection and filtration management measures had a certain bactericidal effect on the SARS-CoV virus (24–26), the conditions were sufficient to create a healthy and safe leisure sports environment (29, 30). These were beneficial for people to concentrate and relax their bodies during leisure sports (33, 34), effectively obtain the effect of leisure sports (19, 72), and achieve the purpose of promoting physical and mental health (19, 72). Therefore, the researchers put forward five hypotheses based on the above inferences and research framework.

Hypothesis 1. Assume that the risk perceptions of the exercise environment for swimming in swimming pools are consistent.
Hypothesis 2. The body anxiety perceptions of swimming in the pool are consistent.
Hypothesis 3. The perception of leisure satisfaction of swimming in swimming pools is consistent.
Hypothesis 4. The physical and mental health perceptions of swimming in the pool are consistent.
Hypothesis 5. Environmental risk has a positive and significant effect on physical anxiety, leisure satisfaction, and physical and mental health.

### Research Tools and Analysis

The study used a mixed approach, with references to environmental risk (46–51), physical anxiety (52–57), leisure satisfaction (58–61), and perceptions of physical and mental health (37, 58–61), and then prepared a preliminary questionnaire. The questionnaire was divided into background information and variables. The background was gender (male, female) and age (under 30, 31–40, 41–50, over 51). Other variables were divided into environmental risk, physical anxiety, leisure satisfaction, and physical and mental health. There were 42 questions in total and were designed with a Likert-style 5-point scale (1 point meant very dissatisfied, 5 points meant very satisfied). After the preliminary questionnaire was edited, according to the Kaiser-Meyer-Olkin (KMO) > 0.06 in the test results, and the *p*-value in the Bartlett test was < 0.01 (*p* < 0.01) (73), α > 0.60. The questionnaire met the standard of good reliability issues (74), and the content of the questionnaire applicable to the subject of this research was reserved for continuous research and analysis.

According to references, edited environmental risk design six questions (47–51). The analysis results showed that the KMO was 0.837, the approximate χ2 value of Bartlett was 970.578, the df was 6, and the significance was *p* < 0.001, which was suitable for factor analysis. The total variance explained by the scale was 67.414%. After factor analysis, good and reliable topics were retained. The alpha coefficient of the total scale was 0.891. After deleting the inappropriate topics, they were four topics reserved at the end, “modifying body movements,” “exercise methods will not cause infection risks,” “good mood,” and “no increase in social costs.” Each topic scored 0.855 to 0.862. According to the above analysis results, it could be known that this questionnaire had good reliability.

Referring to (52–57) literature, 10 questions were designed to edit body anxiety cognition. The analysis results showed that the KMO was 0.683, the approximate χ2 value of Bartlett was 2,634.081, the df was 28, and the significance was *p* < 0.001, which was suitable for factor analysis. The variance explained by the scale was 38.131 and 30.148%, and the total explained variance was 68.28%. After factor analysis, good and reliable issues were retained and named psychological and physical (5 questions) and achievement performance (3 questions). The alpha coefficient of the total scale was 0.851. After deleting the inappropriate items, they were reserved at the end. There were 8 questions in total, including anxiety, tension, physical incoordination, muscle relaxation, stomach pain, performance satisfaction, achievement of goals, and surpassing level. Each question was 0.809–0.849. According to the above analysis results, it could be known that this questionnaire had good reliability.

Referring to the literature (58–61), a total of nine questions were designed to edit the cognition of leisure satisfaction. The analysis results showed that the KMO was 0.683, the approximate χ2 value of Bartlett was 2,634.081, the df was 28, and the significance was *p* < 0.001, which was suitable for factor analysis. The variance explained by the scale was 38.131 and 30.148%, and the total explained variance was 68.28%. After factor analysis, good and reliable issues were retained and named as epidemic prevention measures (3 questions), water quality and environment (3 questions), and leisure effects (2 questions). The alpha coefficient of the total scale was 0.908. After deleting the inappropriate topics, eight questions were finally retained 8 questions in total, including wearing masks, epidemic prevention distance, source of swimmers, personal epidemic prevention and hygiene literacy, clean water quality, clean facilities, environment, exercise ability, and completed actions. Each question scored 0.894–0.897. According to the above analysis results, it could be known that this questionnaire had good reliability.

Referring to the literature (62–65), a total of 17 questions were designed to edit physical and mental health cognition. The analysis results showed that the KMO was 0.756, the approximate χ2 value of Bartlett was 8,613.85, the df was 136, and the significance was *p* < 0.001, which was suitable for factor analysis. The variance explained by the scale was 38.131 and 30.148%, and the total explained variance was 68.28%. After factor analysis, good and reliable issues were reserved and named as psychological feelings (5 questions), mental state (5 questions), physical conditions (3 questions), and negative thoughts (4 questions). The alpha coefficient of the total scale was 0.908. After deleting the inappropriate topics, the remaining topics were facing environmental pressure, feeling scared, unsatisfactory performance, not interested in life, unable to make good use of time, head pressure, fatigue, pain sensitivity, back tightness, insomnia, stomach pain, eating disorders, body tension, easy tantrums, worthless, life burnout, and suicidal escapism, a total of 17 questions. Each question scored 0.894–0.897. According to the above analysis results, it could be known that this questionnaire had good reliability.

According to the above analysis results, there were 37 questions left in the background disguised, environmental risk (4 questions), physical anxiety (8 questions), leisure satisfaction (8 questions), and physical and mental health (17 questions). Finally, three experts were asked to check the content validity, confirm the content of the questionnaire, and confirm the completion of the formal questionnaire, and then investigation and analysis were conducted.

### Research Scope and Object

Meizhou City is located in the mountainous area of Guangdong Province. Since the outbreak of the epidemic in 2019, the epidemic control mechanism has been continuously adjusted. Although there are still sporadic outbreaks of overall infection cases in various provinces, a number of strict control measures have been carried out on the epidemic situation in the case areas. So far, they have been effectively controlled, and no large-scale infection incidents have occurred (75). In addition, the local latitude is high, the climate change is small, and the people's willingness to invest in swimming is relatively high.

Therefore, the research team limited the sampling to medical workers who were still engaged in swimming activities during the epidemic. Taking Meizhou City as the core of the research and investigation scope, the interest sampling method was used to collect the basic sample size. At the same time, the snowball sampling method was used to invite people who had agreed to be surveyed to assist in recommending, and other samples were collected through the online questionnaire platform, which was radiated and diffused outwards, and finally, 840 samples were obtained.

In addition, the interview process invited six experts in decision analysis, public health, and leisure sports management, as well as long-term swimmers, the general public, and industry players, to obtain the interviewee's consent to the interview by using the video system and telephone calls. Then the semi-structured interview method was used to conduct the survey, and opinions were put forward on the questionnaire analysis and the results. The interview subjects and interview topics are described in Table 1.

**Table 1:** Respondent's background information and an overview of the interview outline.

**Identity**	**Gender**	**Residence and sports time/years of work experience**	**Identity**	**Gender**	**Residence and sports time/years of work experience**
Swimmer	Male	31	Professor	Female	15
Swimmer	Female	41	Entrepreneur	Female	30
Professor	Male	20	Entrepreneur	Male	10
Professor	Male	16	People	Male	40
**Construct**	**Issues**				
Impact of tourism development	1. Do you agree that during the exercise in the swimming pool, the sports environment still feels the threat of the epidemic? What parts? What is the reason? 2. Do you agree that physical and mental health will be threatened during exercise in the swimming pool? Which parts? Why? 3. Do you agree that leisure satisfaction is affected by exercising in the swimming pool? Which parts? Why? 4. Do you agree that your physical and mental health will be affected by exercising in the swimming pool? Which parts? Why?				

### Ethical Considerations

This study was conducted using a mixed-type study, with Meizhou City as the scope. The standard for collecting questionnaire samples is local people with medical work status who have continued to swim since the beginning of the epidemic. Investigators produced a brief description of the swimming pool safety research report, briefed respondents or asked respondents to read the content during the sampling period, and then began the survey. Respondents, therefore, had an understanding of the research topic and had relevant professional competence or personal experience. Therefore, all respondents agreed to and understood the purpose of this research and then cooperated with the presentation of anonymous data and under the condition of knowledge, recorded and collected data, and agreed to cooperate with the provision of relevant data for data collection. The research design and data collection process were carried out under the standards of fairness, impartiality, and openness. Therefore, the researchers believed that the research conformed to ethical standards and did not need to apply for ethical certification (75, 76).

However, during the research and investigation period, there were still some cases of epidemic transmission in China. In order to avoid the risk of infection, the research and investigation team only took the local area of Meizhou City as the on-site distribution scope. Considering the limitations of resources such as human resources, material resources, and funds of the team, as well as factors such as the time node of swimming participation and the location of swimming pools for assisting pushers, the above limitations might affect the total number of samples and analysis results. Therefore, if the results of this study were insufficient, they would be listed as recommendations for follow-up research investigations in the hope that follow-up researchers would strengthen them.

## Analysis and Discussions

The study used basic statistics, *t*-test, ANOVA, and PPMCC test methods to analyze the related issues among background information, environmental risks, physical anxiety, leisure satisfaction, physical and mental health cognition, and variables in 840 samples.

The analysis revealed that among the 840 samples, 276 (32.2%) were male and 580 (67.8%) were female. Among the age groups, 304 (35.5%) were under the age of 30, 376 (43.5%) were 31–40 years old, 152 (17.8%) were 41–50 years old, and 28 (3.3%) were over 51 years old. It could be seen that most of the samples tested were women, and the least were men; people under the age of 40 were the majority, and those over the age of 51 were the least.

### Analysis of People's Sports Environment Risk, Physical Anxiety, Leisure Satisfaction, Physical and Mental Health Cognition

The analysis used basic statistics, *t*-test, and ANOVA to analyze the environmental risks, physical anxiety, leisure satisfaction, and physical and mental health cognition of people swimming in swimming pools under the epidemic and verify Hypotheses 1–4. The analysis found that the modified action (3.95) was the highest in the risk environment, and the exercise mode did not have the lowest risk of infection (3.77). In physical anxiety, tension (3.52) and performance satisfaction (3.3) were the highest, muscle relaxation (3.0) and goal achievement (3.14) were the lowest. Leisure satisfaction was the highest in terms of distance from epidemic prevention (3.61), clean water (3.6), exercise ability (3.58) while wearing a mask (3.55), clean facilities (3.55), and completing actions (3.55) were the lowest. Physical and mental health was the highest with the inability to use time well (2.79), insomnia (2.87), body tension (2.79), and easy to lose temper (2.58). Dissatisfaction with performance (2.65), head pressure (2.41), stomach pain (2.48), and no value (2.33) were the lowest. The above analysis results confirmed that Hypotheses 1–4 were invalid.

In the follow-up analysis, gender was insignificant in environmental risk and leisure satisfaction (*p* > 0.01). In terms of physical anxiety, tension, limb incoordination, achievement of goals, and transcendence were significant (*p* < 0.01), and the others were not significant. Men felt high in tension and limb incoordination, and women felt high in transcendence level. In terms of physical and mental health cognition, facing environmental pressure, performance dissatisfaction, head pressure, fatigue, insomnia, eating disorder, and no value were significant (*p* < 0.01), and the others were not significant. And men had higher feelings of head pressure, fatigue, and insomnia, while women had higher feelings of environmental stress, unsatisfactory performance, eating disorders, and worthlessness.

In addition, subjects of different ages toward the issues in good moods, showing satisfaction, reaching goals, exceeding levels, facing environmental stress, feeling scared, head pressure, fatigue, pain sensitivity, back tightness, insomnia, stomach pain, eating disorders, and life burnout were significant (*p* < 0.01), and others were not. In addition, those under the age of 30 were more sensitive to the performance of physical anxiety, exceeding the level, as well as physical and mental health issues such as head pressure, pain sensitivity, stomach pain, eating disorders, and life burnout. Those aged 31–40 were more sensitive to physical anxiety, performance satisfaction, achievement of goals, and exceeding levels, as well as physical and mental health issues such as fear, pain sensitivity, back tightness, stomach pain, eating disorders, and life burnout. Those aged 41–50 were more sensitive to environmental stress issues in achieving physical and mental health. People over 51 years old were satisfied with their performance in physical anxiety, and their physical and mental health were more sensitive to fear, head pressure, pain sensitivity, back tightness, and insomnia.

It could be seen that most people believed that swimming could modify body movements, but there was still a risk of infection. Although the performance of the exercise was satisfactory, the body was still tense, the muscles could not relax, and the exercise goal could not be achieved. Even if the distance of epidemic prevention and the clean water were reassuring, the exercise ability could be improved. However, problems such as wearing masks by the swimmers and the cleanliness of the facilities remained, and the swimmers could not complete the movements, resulting in a lack of time, insomnia, and physical tension among the people's physical and mental health. These caused different genders to have cognitive differences in physical anxiety and physical and mental health cognition. Different ages also had different views on physical anxiety and physical and mental health cognition. As shown in Table 2.

**Table 2:** Analysis of people's sports environment risk, physical anxiety, leisure satisfaction, physical and mental health cognition.

			**M**	**Rank**	**Gender**					**Age**					
					**Male**		**Female**		***p***	**Under 30 (M)**	**31–40** **(M)**	**41–50** **(M)**	**Over 51** **(M)**	***p***	***Post-hoc***
					**M**	**SD**	**M**	**SD**							
Sports environment risk		Modify body movements	3.95	1	3.93	0.731	3.96	0.692	0.601	4.01	3.90	3.95	4.00	0.337	NA
		No risk of infection	3.77	4	3.68	0.837	3.82	0.765	0.084	3.84	3.73	3.76	3.64	0.542	NA
		Good mood	3.88	2	3.83	0.779	3.91	0.682	0.066	3.97	3.85	3.76	4.00	0.002*	NA
		No social cost	3.85	3	3.87	0.733	3.84	0.717	0.828	3.89	3.80	3.89	3.93	0.500	NA
Physical anxiety	Mental and physical	Anxiety	3.37	3	3.67	0.738	3.23	0.902	0.651	3.25	3.35	3.63	3.43	0.094	NA
		Nervous	3.52	1	3.97	0.639	3.30	0.929	0.000*	3.36	3.51	3.68	4.57	0.101	NA
		Limb incoordination	3.48	2	3.71	0.642	3.37	0.997	0.000*	3.29	3.52	3.55	4.57	0.028	NA
		Muscle relaxation	3.00	5	2.81	0.892	3.08	1.029	0.804	3.00	3.25	2.47	2.43	0.071	NA
		Stomachache	3.03	4	2.81	1.084	3.14	1.069	0.209	2.93	3.34	2.50	2.86	0.263	NA
	Achievement performance	Performance satisfaction	3.30	1	3.25	0.772	3.32	0.695	0.168	3.30	3.47	2.71	4.14	0.000*	Under 30, 31–40, over 51 > 41–50
		Achieve goals	3.14	3	3.16	1.048	3.14	0.731	0.000*	3.21	3.33	2.45	3.71	0.000*	31–40 > under 30, 41–50; over 51 > 41–50
		Beyond the level	3.21	2	3.09	1.091	3.27	0.800	0.001*	3.11	3.42	2.82	3.71	0.000*	Under 30, 31–40 > 41–50, over 51; 41–50 > over 51
Leisure satisfaction	Epidemic prevention measures	Wear mask	3.55	3	3.56	1.190	3.54	1.205	0.927	3.48	3.53	3.72	3.57	0.759	NA
		Epidemic prevention distance	3.61	1	3.59	1.182	3.63	1.170	0.872	3.58	3.65	3.58	3.71	0.196	NA
		Source of bathers	3.60	2	3.62	1.166	3.59	1.194	0.940	3.64	3.52	3.71	3.71	0.523	NA
	Water quality	Personal epidemic prevention literacy	3.59	2	3.62	1.161	3.57	1.139	0.978	3.53	3.56	3.67	4.07	0.230	NA
		Water quality	3.60	1	3.63	1.203	3.59	1.237	0.686	3.55	3.56	3.75	3.86	0.201	NA
		Clean facility	3.55	3	3.58	1.213	3.53	1.153	0.406	3.51	3.49	3.71	3.71	0.360	NA
	leisure effect	athletic ability	3.58	1	3.55	1.215	3.59	1.217	0.971	3.57	3.53	3.63	4.00	0.038	NA
		Complete the action	3.55	2	3.60	1.199	3.53	1.123	0.346	3.61	3.44	3.71	3.64	0.878	NA
Physical and mental health	Psychological feeling	Facing environmental pressure	2.75	2	2.55	1.061	2.85	0.867	0.000*	2.72	2.87	2.34	3.71	0.000*	41–50 < under 30, 31–40, over 51
		Feeling scared	2.67	4	2.51	0.976	2.74	0.902	0.049	2.49	2.86	2.37	3.71	0.000*	31–40 > under 30, 41–50, over 51; over 51 > under 30, 31–40, 41–50
		Unsatisfactory performance	2.65	5	2.64	1.053	2.66	0.866	0.003*	2.42	2.86	2.34	4.14	0.072	
		Not interested in life	2.72	3	2.38	0.803	2.88	0.995	0.848	2.67	2.94	2.32	2.57	0.184	
		Can't make good use of time	2.79	1	2.71	0.906	2.82	0.939	0.126	2.57	2.98	2.50	4.14	0.036	
	Mental condition	Head pressure	2.41	5	2.16	1.089	2.53	0.873	0.005*	2.16	2.87	1.66	3.14	0.010*	Under 30 > 41–50, over 51; 31–40 > under 30, 41–50; over 51 > under 30, 41–50
		Tired	2.44	4	2.61	1.014	2.36	0.862	0.003*	2.29	2.66	2.11	3.00	0.000*	31–40 > under 30, 41–50; over 51; over 51 > under 30, 41–50
		Pain sensitivity	2.58	3	2.51	1.102	2.61	1.047	0.505	2.50	2.77	1.97	4.14	0.000*	Under 30 > 41–50, over 51; 31–40 > 41–50; over 51 > under 30, 31–40, 41–50
		Tight back	2.62	2	2.59	1.150	2.63	1.065	0.083	2.34	2.80	2.37	4.57	0.002*	31–40 > under 30, 31–40, 41–50; over 51 > under 30, 31–40, 41–50
		Insomnia	2.87	1	3.00	1.232	2.81	0.948	0.000*	2.75	2.86	2.84	4.57	0.000*	over 51 > under 30, 31–40, 41–50
	Physical conditions	Stomachache	2.48	3	2.19	0.892	2.62	0.942	0.215	2.36	2.85	1.92	2.00	0.000*	under 30 > 41–50, over 51; 31–40 > under 30, 41–50, over 51
		Eating disorders	2.63	2	2.46	1.141	2.70	1.026	0.001*	2.46	2.92	2.42	1.57	0.000*	31–40 > under 30, 41–50, over 51; under 30 > over 51
		Limbs tense	2.79	1	2.70	0.860	2.83	0.936	0.163	2.64	3.02	2.53	2.57	0.530	
	Negative thoughts	Easy to lose temper	2.58	1	2.17	0.903	2.78	0.827	0.063	2.32	2.88	2.42	2.43	0.080	
		No value	2.33	3	2.12	1.047	2.43	0.870	0.003*	2.17	2.61	2.08	1.57	0.136	
		Life burnout	2.46	2	1.96	0.973	2.70	1.006	0.661	2.49	2.73	1.92	1.57	0.004*	Under 30, 31–40 > 41–50, over 51
		Suicide to escape reality	2.28	4	1.68	0.928	2.56	1.038	0.045	2.22	2.70	1.47	1.57	0.089	

*P < 0.01.

### Correlation Analysis of Physical Anxiety, Leisure Satisfaction, and Physical and Mental Health Cognition of Sports Environmental Risks

The PPMCC test method was used to analyze the impact of environmental risks on physical anxiety, leisure satisfaction, and physical and mental health cognition of people who still engage in swimming during the epidemic. The analysis showed that environmental risk had a positive and significant positive effect on physical anxiety (0.299) and leisure satisfaction (0.299), and *p* < 0.01, but had no significant effect on physical and mental health cognition. Among them, environmental risk cognition had the greatest influence on achievement performance (0.298) and leisure effect (0.370); it had less influence on psychology and physiology (0.253) and water quality environment (−0.126). And found that environmental risk cognition and water quality environment showed a negative significance. The results verified that Hypotheses 5-1 and 5-2 were valid, but 5-3 was not.

It could be seen that environmental risk had a positive and significant influence on physical anxiety and leisure satisfaction, among which achievement performance and leisure effect had the greatest influence, and psychological and physiological influences were the least. Moreover, when the awareness of environmental risks increased, the public's satisfaction with the water quality and environment of the swimming pool decreased. As shown in Table 3.

**Table 3:** Correlation analysis of sports environmental risk on physical anxiety, leisure satisfaction, and physical and mental health cognition.

**Sports environment risk**	**Physical anxiety**			**Leisure satisfaction**			
	**Overall facet**	**Mental and physical**	**Achievement performance**	**Overall facet**	**Epidemic prevention measures**	**Water quality**	**Leisure effect**
Overall facet	0.299**	0.253**	0.298**	0.137**	−0.030	−0.126*	0.370**
Modify body movements	0.013	0.001	0.028	0.023	−0.022	0.059	0.035
No risk of infection	0.036	0.010	0.072	0.000	−0.034	0.054	0.009
Good mood	0.003	−0.020	0.050	0.048	0.043	0.093	0.003
No social cost	−0.017	−0.025	0.000	0.045	−0.006	0.101	0.044
**Sports environment risk**	**Physical and mental health**						
	**Overall facet**	**Psychological feeling**	**Mental condition**	**Physical conditions**	**Negative thoughts**		
Overall facet	−0.030	0.128**	−0.046	−0.089	−0.136**		
Modify body movements	−0.009	0.012	−0.009	−0.019	−0.022		
No risk of infection	−0.025	−0.003	−0.037	−0.044	−0.013		
Good mood	0.034	0.055	0.028	0.023	0.010		
No social cost	−0.011	0.008	−0.010	−0.032	−0.015		

* *p < 0.05;*

** *p < 0.01*.

### Discussions

#### Analysis of Background

We believed that although swimming was a whole-body exercise, there were various swimming styles, and the exercise intensity of different swimming styles varied greatly. Swimming could strengthen cardiopulmonary function, effectively improve health (19, 20), help women maintain their posture, and provide people under 40 with sufficient exercise effects. In addition, most medical workers over the age of 51 work as supervisors or important units, and the medical work has heavy tasks. As a result, medical staff over 51 years old have less leisure time and less willingness to go out to swim. Therefore, under the epidemic environment, the majority of people who were still able to continue to participate in swimming were healthcare workers under the age of 40, and women were more willing to engage in swimming than men.

We believed that enterprises should strengthen the publicity and image of epidemic prevention measures and strengthen people's confidence in the epidemic prevention measures of swimming venues. Medical institutions could cooperate with enterprises nearby to provide medical personnel with a swimming and sports environment. Competent agencies could encourage medical personnel to plan swimming exercises to improve physical and mental health. All these measures would improve the willingness of men to participate and the willingness of medical staff of other ages to swim.

#### Analysis of Environmental Risk of Infection, Physical Anxiety, Leisure Satisfaction, and Physical and Mental Health of Swimmers

The research deduced that under the influence of the epidemic, public transportation and open environments were still at risk of infection (3). Therefore, people reduced their willingness to take public transportation and increased the travel time to the swimming pool. This led most people to believe that the existing exercise time was squeezed. However, the exercise time was insufficient, resulting in insufficient exercise, so the body was still tense, unable to solve the problems of insomnia, limb tension, and easy tantrums. In addition, due to China's current measures to clear and prevent blockages, the government used mobile phones to control personnel dynamics. Under the long-term epidemic prevention propaganda, the public's awareness of self-epidemic prevention and keeping distance gradually increased. In addition, the swimming pool used sodium hypochlorite for water quality management and environmental cleaning, and its items could effectively reduce the activity of the new coronavirus (25, 26), coupled with the repeated water filtration mechanism, to ensure the hygiene and quality of swimming pool water. Therefore, most healthcare workers believe that the swimming pool is in line with their anti-epidemic benefits and is a safe and healthy leisure sports environment that can help relieve tension. The exercise investment could slow down, people could maintain their own pace to modify their swimming style and technique, and swimming could increase swimming ability and life performance.

In addition, in a low-risk environment, the mentality was stable. The exercise investment could slow down, people could maintain their own pace to modify their swimming style and technique, and swimming could increase swimming ability and life performance.

Therefore, most healthcare workers believed that swimming could modify body movements, but there was still a risk of infection. Although the performance of the exercise was satisfactory, the body was still tense, the muscles could not relax, and the exercise goal could not be achieved. Even if the distance of epidemic prevention and the clean water were reassuring, the exercise ability could be improved. However, issues such as wearing masks by swimmers, cleaning auxiliary facilities, or returning to the territory had not been perfected and could not be implemented. Ultimately, physical and mental health still produced feelings of lack of time, insomnia, and limb tension.

Next, different gender dimensions were explored. Due to the degree of exercise investment in different genders, there were certain differences in exercise time planning and immunity (75). Due to the differences in innate physiological structure, men had high exercise ability and high intensity, but their metabolism was slow, which led to muscle tension. In addition, the tasks of male healthcare workers will be heavier, increasing the pressure burden. As a result, it is difficult for them to recover from fatigue, sleep, and physical and mental stress.

Women were more delicate in their minds and were more able to find measures to disperse the stress and maintain their level of exercise when facing pressure. This is also the main reason why women are valued as healthcare workers. However, due to the diversity of swimming pool activity spaces and participants' identities, as well as different sanitation and epidemic prevention literacy, the risk of infection increased. When women engaged in sports in an uncertain sports environment, they easily lost the existing rhythm of life, disrupted existing plans, and affected life behavior and sports performance. As a result, the daily routine was changed, the way of eating was affected, the frustration and loss in life increased, and the physical and mental health were damaged.

Therefore, different genders had cognitive differences in physical anxiety physical and mental health cognition. Among them, men reported higher levels of tension, physical incoordination, head pressure, fatigue, and insomnia, while women experienced higher levels of issues such as transcendence, environmental stress, unsatisfactory performance, eating disorders, and worthlessness.

Finally, different age levels were discussed. Due to differences in reaction ability, virus resistance, life behavior, and mobility at different ages, different ages had different views on physical anxiety and physical and mental health cognition when faced with an infected sports environment (77, 78). Among them, because 31–40-year-old healthcare workers were in the process of physical maturity and strong strength, and the current work stage might be starting or reaching an important growth moment, there was an urgent need for ways to relieve stress. However, although swimming pools' pool management methods and environmental cleaning measures had a certain protective power, the epidemic situation continued to appear, and there was a gap between the public's personal epidemic prevention awareness and self-protection awareness, which was prone to epidemic prevention gaps. In addition, there was a lot of pressure from work or life, but it was impossible to relieve the pressure through sufficient exercise time and amount of exercise. As a result, people's physical and mental health in the 31–40-year-old age group had not been improved.

Furthermore, most people aged 41–50 had accumulated many years of experience in life, work, family care, and personal physical and mental health management (78). Most healthcare workers had a wealth of experience and knowledge at this age. Therefore, although there might be concerns that the current surrounding living environment was still full of the risk of virus infection, reducing the amount of exercise could not achieve the expected exercise goals. However, in the face of the epidemic environment and risks, people of this age group could plan their own health and epidemic prevention techniques and strategies based on their past experience and knowledge.

In addition, although people over the age of 51 were in the peak period of life, work, family, and self-health management (79), they might be about to stabilize or decline in another life stage (77, 80). However, at the same time, they were also gradually facing a state of physical exercise ability, self-health defense function, and mechanism gradually failing (78). Coupled with the impact of the epidemic, the convenience of transportation had disappeared, and the risk to the living environment had increased (3). As a result, people over the age of 51 could use their rich personal experience to find suitable alternative exercise methods and self-health management strategies under the epidemic situation to achieve the goal of stable life and exercise quality. However, due to the heavy medical work during the epidemic, coupled with the gradual failure of the individual's self-health defense function and mechanism, and the fear of virus infection, physical and mental health would be threatened, causing tension in the head, shoulders, neck, back, and other body parts and affecting sleep quality.

Therefore, different ages had different views on physical anxiety and physical and mental health cognition. Among them, 31–40-year-olds were more sensitive to issues such as satisfaction with physical anxiety, achievement of goals, surpassing levels, and physical and mental health, as well as fear, pain sensitivity, back tightness, stomach pain, eating disorders, and life burnout. People aged 41–50 were more sensitive to issues such as meeting their goals and facing environmental pressures on their physical and mental health. Most healthcare workers over the age of 51 were more sensitive to issues such as physical anxiety, fear of physical and mental health, head pressure, pain sensitivity, back tightness, and insomnia.

We suggested that a safe and proper transportation epidemic prevention mechanism should be planned, and appropriate transportation routes should be established. The study also recommended designing swimming and physical exercise mechanisms in line with men, improving swimmers' epidemic prevention and hygiene literacy, and providing women with a safe swimming environment. Then, according to the needs of people aged 31–50, appropriate exercise prescriptions should be well planned, and exercise measures that could eliminate head and body fatigue, promote blood circulation, and improve the quality of sleep should be well designed to improve the health problems of people over 51 years old. The researchers believe that these measures can stabilize the health of healthcare workers and achieve the purpose of improving physical and mental health.

#### Correlation Analysis of Sports Environmental Risk on Physical Anxiety, Leisure Satisfaction, and Physical and Mental Health Cognition

This study inferred those studies had confirmed that the use of sodium hypochlorite for disinfection and cleaning in swimming pools could inhibit the activity of SARS-CoV-2 and achieve disinfection effects (27–30, 42), and swimming could help improve physical and mental health and strengthen cardiopulmonary function (19, 20). However, since the SARS-CoV-2 virus is still raging, Omicron has a low fatality rate and a high risk of infectivity. The emergence of mutant strains has the risk of re-infection, resulting in increased resistance to people trying to restore order in life and leisure. Coupled with the limited sports environment and people's different personal epidemic prevention sensitivities, hygiene, and environmental literacy, there might still be infection risks in swimming pools. This would lead to a reduction in the swimming people's leisure performance and final sports effect, and the expected leisure benefits and sports goals could not be obtained.

Therefore, the public's environmental risk cognition had a positive and significant correlation with physical anxiety and leisure satisfaction cognition. Among them, achievement performance and leisure effect were most influenced by environmental risk cognition. When the environmental risk cognition was higher, swimming pool water quality and environmental satisfaction would decrease.

We suggested that if the swimming pool environment could maintain the existing quality of disinfection and filtration, medical workers could first be provided with different leisure sports measures to achieve the purpose of improving physical and mental health. It would be a better decision if the swimming pool staff could repeatedly remind other swimmers to abide by the epidemic prevention regulations in the swimming pool, regulate the safe exercise distance, and continue to use sodium hypochlorite to increase disinfection in other swimming pool spaces.

## Conclusions and Suggestions

Medical workers are able to maintain the effect of exercise and maintain their health by strengthening the measures of water filtration and disinfection, enhancing the confidence in the swimming pool environment and epidemic prevention and control because the operators control the living footprints of swimmers. However, the public's awareness of epidemic prevention, hygiene awareness, and environmental literacy are different, causing people to have doubts about the effectiveness of epidemic prevention in bathrooms, toilets, and parking spaces. These lead effectively improve the environmental risk perception of medical personnel, reduce anxiety, improve leisure benefits, and achieve the goal of promoting physical and mental health. Female medical workers believe that the current epidemic prevention effect is still damaged, and male medical workers believe that the current sports benefits of going swimming are not good. Those aged 31–50 feel that exercise program options are few and ineffective, and those over 51 feel they still have problems with fear, head pressure, pain sensitivity, back tightness, and insomnia. The resulting environmental risk perceptions are positively correlated with body anxiety and leisure satisfaction perceptions and affect achievement performance and leisure outcomes. The higher the environmental risk, the more pronounced the impact of pool water quality and the effectiveness of environmental maintenance on healthcare workers.

We suggested that a safe and proper transportation epidemic prevention mechanism should be planned, and appropriate transportation routes should be established. Swimming pools can plan swimming exercise mechanisms for different genders and ages, strengthen publicity and epidemic prevention mechanisms, and improve the cooperation of other swimmers in epidemic prevention. These strategies will effectively improve the environmental risk perception of medical personnel, reduce anxiety, improve leisure benefits, and achieve the goal of promoting physical and mental health. Based on the above analysis and inference results, the study suggests research on the government, venues, the public, and future studies.

### About the Government

Plan a safe and proper transportation epidemic prevention mechanism, and establish suitable transportation routes. Improve people's epidemic prevention and hygiene literacy, and provide a safe swimming environment.

### About the Pool Venue

The swimming pool environment should maintain the existing disinfection and filtration quality, improve the epidemic prevention measures, add slogans or voice announcements to remind the public to abide by the epidemic prevention regulations in the swimming pool sports environment, and then standardize the safe exercise distance in the existing swimming sports space. Or, in the building space, add other sports facilities, and use the disinfection effect of sodium hypochlorite to maintain the cleanliness of the sports space and facilities.

### About the Healthcare Workers

Improve environmental literacy, follow the epidemic prevention measures of the government and sports venues, jointly maintain the sports environment, maintain the effectiveness of epidemic prevention, and avoid becoming an epidemic prevention gap.

### Suggestions for Future Research Directions

The researchers suggest extending the discussion to different occupations or identities of people, as well as other issues. It is expected that follow-up researchers will develop swimming exercise prescriptions or other physical exercise mechanisms in line with 31–50-year-old males and design exercise measures that can eliminate head and body fatigue, promote blood circulation, and improve sleep quality for people over 51 years old. The research gaps will be completed.

## Data Availability Statement

The original contributions presented in the study are included in the article/supplementary material, further inquiries can be directed to the corresponding author/s.

## Ethics Statement

Ethics review and approval/written informed consent was not required as per local legislation and institutional requirements.

## Author Contributions

Z-CL and Y-HT: conceptualization and funding acquisition. Z-CL: methodology, software, visualization, resources, and project administration. Z-CL, C-HT, H-HL, and Y-HT: validation. C-HT: formal analysis. H-HL: investigation, data curation, writing—original draft preparation, and supervision. Y-HT: writing—review and editing. All authors have read and agreed to the published version of the manuscript. All authors contributed to the article and approved the submitted version.

## Conflict of Interest

The authors declare that the research was conducted in the absence of any commercial or financial relationships that could be construed as a potential conflict of interest.

## Publisher's Note

All claims expressed in this article are solely those of the authors and do not necessarily represent those of their affiliated organizations, or those of the publisher, the editors and the reviewers. Any product that may be evaluated in this article, or claim that may be made by its manufacturer, is not guaranteed or endorsed by the publisher.
